# Influence of Ethanol on Pitting Corrosion Behavior of Stainless Steel for Bioethanol Fermentation Tanks

**DOI:** 10.3389/fchem.2020.00529

**Published:** 2020-06-24

**Authors:** Yiting Wan, Yangting Sun, Dingzhou Cai, Liqiang Yin, Nianwei Dai, Longlin Lei, Yiming Jiang, Jin Li

**Affiliations:** ^1^Corrosion Lab, Department of Materials Science, Fudan University, Shanghai, China; ^2^Institute of Materials, China Academy of Engineering Physics, Mianyang, China

**Keywords:** stainless steel, ethanolic solutions, pitting corrosion, potentiodynamic polarization, morphology

## Abstract

The role of ethanol (C_2_H_5_OH) in pitting corrosion behavior of AISI 316L austenitic stainless steel was investigated in aqueous ethanolic solution with chloride. The pitting susceptibility and surface morphology of 316L in a series of ethanol-containing solutions were examined using X-ray photoelectron spectroscopy (XPS), optical microscopy with 3D stitching, immersion tests, and potentiodynamic polarization measurements. Results demonstrated that the ethanol concentration impacted little on the passive film stability while it dramatically influenced the pitting corrosion susceptibility. Corrosion rate of 316L after immersion tests first increased and then decreased as the concentration of ethanol increased from 0 to 10 M in ferric chloride solution. This, however, did not correspond to the breakdown potential which directly decreased from 489 to 249 mV as the water concentration decreased in ethanolic NaCl solutions. The pits density after both immersion and electrochemical tests showed that the initiation of pitting in ethanolic solution tended to occur at multiple points at the same time. The synergy effect on pitting behavior of hydrolysis enhancement and solubility reduction of metal cations due to the introduction of ethanol has also been discussed.

## Introduction

Bioethanol has been widely recognized as a promising renewable sustainable biofuel to replace diminishing petroleum-based fuel in existing combustion motor system. Regular crops such as corn and sugarcane, due to their chief value in food reserves, are unable to meet the increasing worldwide demand of fuel-grade ethanol. Thus, agriculture wastes, particularly lignocellulosic biomass like straw and microalgae, with competitive cost and abundant stock are potential raw materials for bioethanol. The key factor for gradual replacement of feedstocks is to adjust production process owing to different degradability of sugar, starch, and cellulose. With almost the same process flow, lignocellulosic ethanol production differs a lot on pre-treatment and fermentation. Lignocellulosic ethanol production prefers acid-based methods in one-step fermentation process to enzyme-cocktail based methods, which is commonly used in crop-based ethanol production but introduces fewer corrosive contaminants such as chlorides and sulfuric acid (Sarkar et al., 2012). Currently, some lignocellulosic ethanol is still fermented in traditional vessels designed for crop-based bioethanol focusing on mechanical strength but not on corrosion resistance (Torsner, 2010). Before the biological fermentation methods become the best choice, various corrosion problems, such as pitting, crevice corrosion, and stress corrosion crack, still exist in ethanol production process. For fear of corrosion accident in bioethanol production, coating was suggested to prevent corrosion of steels in ethanolic environment (Radi et al., 2019). Coating is a widely used method to enhance corrosion resistance. Blunt materials such as epoxy (Bisht et al., 2017), graphite (Wang et al., 2019), alumina (Sun et al., 2019), and polyaniline (Lei et al., 2020) are often recommended. However, there are still many difficulties in using this method in large tanks. Therefore, it is necessary to study the corrosion behavior of traditional stainless steel for bioethanol fermentation tanks, such as AISI 316L stainless steel, which is still widely used in the production of bioethanol, in ethanolic solutions.

Some research works have been done to understand corrosion behavior of metals in various organic media (Ekilik and Grigor'ev, 2002; Newman, 2008; Soriano and Alfantazi, 2016). Organic type (Hronsky, 1981), solution viscosity (Samide et al., 2015), water content (Calabrese et al., 2018), and pH (Kahyarian et al., 2017) could be the main factors that influence the passivation and corrosion behavior of metals. Due to specificity and importance of methanol in chemical industry, numerous studies concentrated on methanolic systems (Wang et al., 2007, 2016; Hu et al., 2012). Discussion on this issue tended to ascribe localized corrosion of stainless steels in methanolic solution to deterioration of passive film and diffusion rate drop with the higher viscosity of methanol (Ekilik and Grigor'ev, 2002). Pitting potential of stainless steels was regarded as a function of water content in methanol/HCl since the formation of passive layer depends on water activity (Hronsky and Duquette, 1982). Some researches postulated that passivation of stainless steel would fail in anhydrous solutions with water less than 70 mole % (Kelly and Moran, 1990). In addition to the influence of methanol on passive film, dissolution kinetics in pits were activated with increasing water concentration in methanol/NaCl at a given potential (Ramgopal and Amancherla, 2012).

In last 10 years, focus shifted from corrosion behavior of metals in methanolic solution to stress corrosion cracking (SCC) and pitting corrosion of carbon steels and stainless steels in simulated fuel grade ethanol (SFGE) (Gui et al., 2010; Lou et al., 2010; Beavers et al., 2011; Samusawa and Shiotani, 2015; Torkkeli et al., 2015) to clarify reliability of pipelines in transportation of bioethanol with minor impurities such as water and chloride. Chlorides, pH, inclusions, temperature even trace water were found that have obvious effect on SCC and pitting susceptibility in SFGE. Study on this issue paid more attention on the impact of trace impurities on corrosion behavior. Iron (II) acetate, which showed strong solubility in FGE and introduced by acetic acid impurities, was unearthed aggravating the passivation of carbon steel with Raman spectra (Samusawa and Shiotani, 2015). Long-term exposure tests in fuel-grade ethanol (Lou and Singh, 2010) showed that pitting susceptibility of carbon steels increased as the water content (below 5 vol.%) increases while water content more than 10 vol.% reduced pitting corrosion.

Limited research reminded that change in the ethanolic solution chemistry could play a crucial role in the localized corrosion of stainless steel. Some work introduced that typical pitting corrosion only occurred in 20 (v/v%) bioethanol SFGE with certain concentration of trace water (Abel and Virtanen, 2015). Ethanol, as an inefficient hydrotropic agent for water and gasoline, induced water and Cl^−^ to enrich on the surface of matrix. It is noteworthy that the precipitation pH of nickel hydroxide would decrease as the concentration of ethanol increases, which makes hydrolysis of Ni^2+^ more intense (Ho and Van Zee, 2000). While researches in SFGE discussed the effect of water on corrosion behavior, inadequate studies have addressed the localized corrosion behavior of stainless steels in solutions with less than 30 mole % ethanol content, which is more suitable for digging out the influence of ethanol. Otherwise, a common defect of experiment in researches in low-ethanol-content solutions is the use of mass fractions as the unit for concentration of contents, which causes the fluctuation activity of contents like chloride since the density of solutions differs with the concentration of ethanol. Ferreira et al. recorded the electrochemical impede spectra (EIS) of 316L in 1 wt.% H_2_SO_4_ and 0.35 wt.% NaCl solutions with and without ethanol to prove that the passive film of stainless steel was decayed by ethanol (Ferreira et al., 2013), which should have been the influence of the higher effective concentration of H^+^ and Cl^−^.

In the present work, pitting behavior and surface morphology of AISI 316L stainless steel, the material widespread in bioethanol fermentation (Rocha et al., 2012; Lee et al., 2013), is investigated. Since the passivation of stainless steel would fail in anhydrous solutions with water less than 70 mole % (Kelly and Moran, 1990), solutions with ethanol content from 0 to 10 M were applied to ensure that changes of corrosion resistance are not caused by extremely low water content, The goal of the work is to clarify the influence of ethanol on passivation, pitting sensitivity and pits morphology. The results proved that the passive film on stainless steel was slightly deteriorated. At the same time, pitting resistance of 316L was severely aggravated even with a spot of ethanol. The relationships between hydrolysis and solubility of metal cations in ethanol and pits pattern are also discussed.

## Experimental

### Specimens Preparation

Samples used in this work were 316L austenitic stainless steel provided by Baoshan Iron & Steel Co Ltd. The chemical composition of the steel is listed in Table 1. Before testing, the as-received samples were machined into large ones (20 × 30 × 3 mm) and small ones (12 × 12 × 3 mm) for chemical immersion tests and electrochemical measurements, respectively. The small samples for electrochemical measurements were mounted with epoxy resin to expose only front surface of specimen for testing. Before testing, all the specimens were ground with SiC papers gradually from 180 to 2,000 grit, polished with diamond polishing powder in 2.5 μm, rinsed with distilled water, degreased with ethanol and dried in blowing air. After then, the epoxy-sealed specimens were covered with perforated insulating tapes (3M 7413D High Temperature Polyimide Tape) to set testing area to 1 cm^2^. All tests were operated just after normalized preparation process to ensure all the specimens were under the same status.

**Table 1:** Chemical compositions of as received 316L stainless steel.

**Element**	**C**	**Si**	**Mn**	**P**	**S**	**Cr**	**Ni**	**Cu**	**N**	**Mo**	**Fe**
wt.%	0.021	0.46	1.37	0.034	0.001	16.39	10.21	0.16	0.034	2.03	Bal.

### Immersion Tests

The immersion tests started with measuring the weight and size of the samples. The approximate molar concentrations of the solution component used in ferric chloride pitting test in ASTM G48-03 (Designation, 2009) was applied to reduce the content difference caused by the density drop with the increase of ethanol. The samples were immersed at 30°C for 24 h in 0.3 M FeCl_3_ + 0.4 M HCl solutions with 0, 1, 2, 5, or 10 M ethanol, respectively. For each solution, three samples were tested at the same time to ensure data repeatability. Since metal cations have different solubility in ethanolic and non-ethanolic solutions, the volumes of solutions were large enough to keep the metal cations dissolved from precipitation during tests. Once the immersion expired and all the samples rinsed with distilled water and dried in blowing air, samples were weighed, and the corrosion rates (*R*_*c*_) were calculated using the expression as followed:

(1) $$\begin{array}{r} R_{c}=\frac{\Delta m}{S\cdot t} \end{array}$$

where Δ*m* is the weight loss of the sample, *S* is the initial surface area of samples, and *t* is the immersion time.

### Electrochemical Measurements

The electrochemical tests were performed under 30°C in 1 M NaCl solutions with 0, 1, 2, 5, or 10 M ethanol. A special three-electrode cell with a platinum counter electrode and a saturated calomel electrode (SCE) as the reference electrode was applied. All the potentials in electrochemical measurements were referred to SCE in this paper if not explicitly defined. O-ring was used to fix the electrodes into the cell to ensure the ethanol not to escape from the system during tests. Since the solubility of oxygen differs in ethanolic solution and in non-ethanolic solution, test solutions were infused with N_2_ gas before the tests for 30 min to adjust the oxygen dissolved in solutions to a close level for each electrochemical test.

Prior to the potentiodynamic polarization tests, cathodic polarization preconditioning under −900 mV for 2 min was conducted to samples. Then, the samples were stabilized at open circuit for 30 min. The polarization scans were performed at 0.6 V h^−1^, starting at −250 mV below the corrosion potential, scanning toward positive potential, until the response current reached 100 μA cm^−2^ or 1 mA cm^−2^ according to demand. The pitting potential (*E*_*p*_) is defined as the given potential at which the current density reaches 100 μA cm^−2^. Since the solution resistivity differs with the concentration of ethanol, the actual potentials of working electrode were corrected with automatic IR compensation offered by the electrochemical workstation. After the measurements, the surface of the sample was slowly cleaned with deionized water, and then dehydrated in an electric drying oven to reduce the damage to the surface morphology of the sample.

### X-Ray Photoelectron Spectroscopy (XPS) Characterization

XPS spectra were recorded by a PHI5300 instrument with a 14-kV monochromatic Mg radiation source to determine the chemical compositions of the passive films of 316L samples. Since whether the passive film differs a lot in non-ethanolic and ethanolic solutions is the point, the electrolytes used to grow up the passive films were 0.02 M H_3_BO_3_ + 0.005 M Na_2_B_4_O_7_ · 10H_2_O borate buffer solutions with or without 5 M ethanol. To ensure the same initial condition, the working electrode was first cathodically polarized at −0.9 V for 5 min in test solutions. Before the XPS characterization, specimens were stabilized at OCP for 4 h. Just after the stabilization process, XPS spectra was taken. XPS data were fitted to determine the type and concentration of compounds in the films using *Avantage* software.

### Morphology Characterization

A single lens reflex camera was used to record the surface morphology of samples after immersion tests. Surface morphology of samples after electrochemical tests was depicted using an optical microscope (DMM-400C). 3D profile of pits was examined with a 3D microscope with super wide depth of field (VHX-1000). The luminance of the output images from under focus to over focus was interpreted as depth and then stitched into the 3D plots. A circline shadowless lamp was introduced to reduce the pit depth error caused by the different convergence of light at the bottom of pits.

All the pits were counted with particle analysis function in ImageJ after fixed threshold binary processing.

## Results

### Chemical Composition of Passive Films

**Figure 1:**
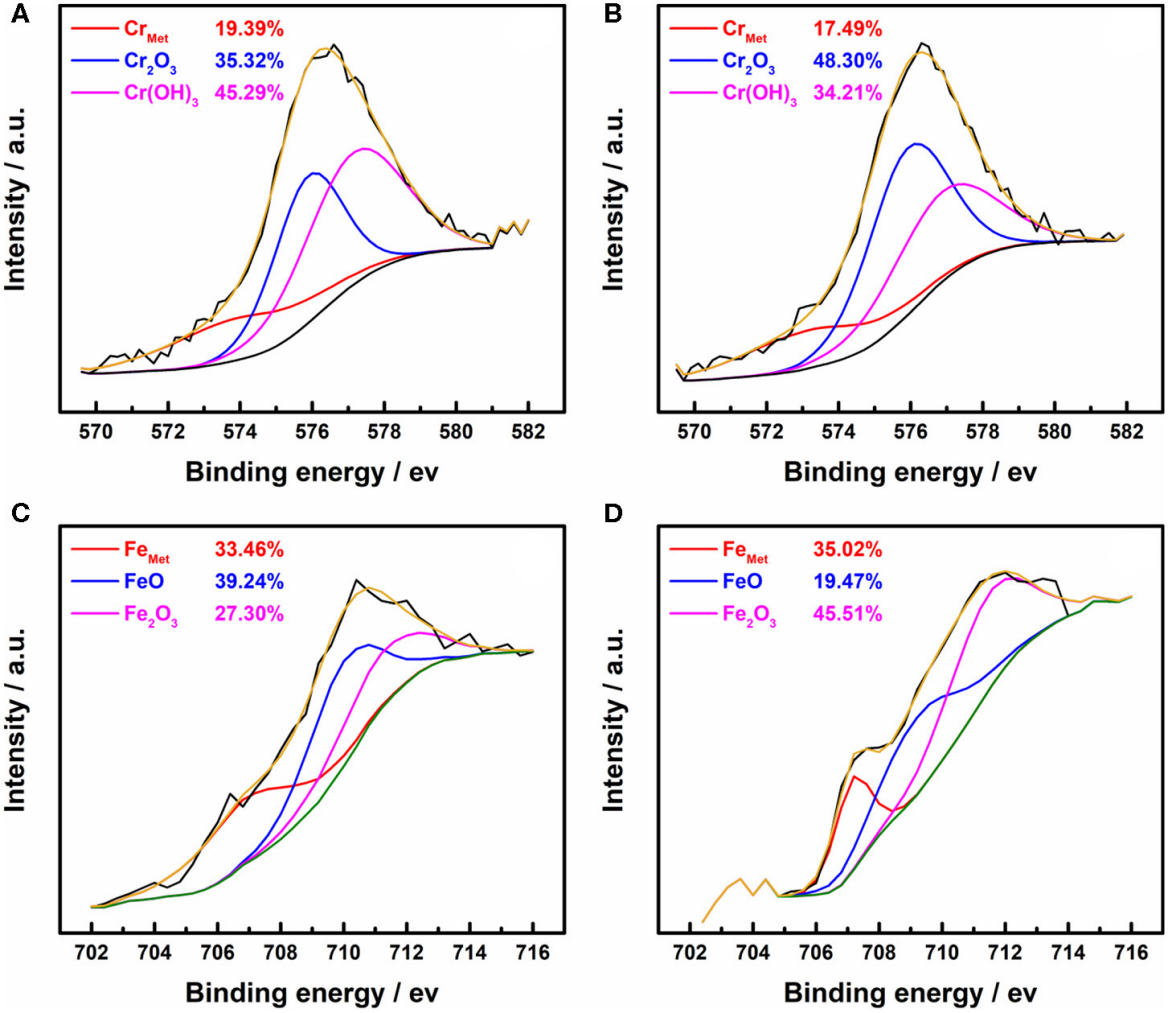
High-resolution XPS spectra of the passive film formed on the 316L specimens in borate buffer solutions. **(A)** Cr 2p_3/2_ in 0 M ethanol, **(B)** Cr 2p_3/2_ in 5 M ethanol, **(C)** Fe 2p_3/2_ in 0 M ethanol, **(D)** Fe 2p_3/2_ in 5 M ethanol.

XPS was employed to clear up how ethanol influences the quality of the passive films on 316L samples. Figures 1A,B shows the high-resolution Cr 2p_3/2_ XPS spectra of 316L samples stabilized in borate buffer solutions with 0 M ethanol and 5 M ethanol, respectively. The area fractions (%Area) are also given for different chemical states of chromium and iron atoms. Three peaks are present in the Cr 2p_3/2_ spectra, including the Cr_met_ (573.1 eV), Cr_2_O_3_ (576.0 eV), and Cr(OH)_3_ (577.2 eV). The area fraction of Cr_2_O_3_ was higher in ethanolic solutions while the area fraction of Cr(OH)_3_ was lower, which could be ascribed to the reduction of water content.

In the Fe 2p_3/2_ spectra in Figures 1C,D, Fe_met_ (706.0 eV), FeO (708.5 eV), and Fe_2_O_3_ (710.0 eV) are present. Since the absence of ethanol in the oxidation-reduction reaction of metal oxides, the area fraction of metal elements does not change significantly. The higher ferric oxide content may be due to the higher solubility of oxygen in the alcohol-containing solution (Shchukarev and Tolmacheva, 1968). As a result, iron oxides with high oxidation states were generated, even if the difference of partial pressure of oxygen was balanced by infusing N_2_ gas.

The atomic contents of the metal oxides, hydroxides species in the passive films of the 316L stainless steel samples have been calculated and summarized in Table 2. For comparison purposes, the total atomic amount of the Cr, Fe, and O elements has been naturalized to 100 at.%. It shows that when the water content is high, the total content of the metal oxides and metal hydroxide changed slightly. Since the content of metal oxides is the main factor of passivation (Okamoto and Shibata, 1970; Saito et al., 1979), the influence of ethanol on the performance of passive film is limited. The result fits to the passivation behavior of stainless steel in low-ethanol-content solutions in the potentiodynamic polarization test (de Anna, 1985).

**Table 2:** Contents of the total metal oxides and metal hydroxides species in the passive films of 316L stainless steel samples calculated from XPS spectra fitting (at. %).

**Ethanol concentration**	**0 M**	**5 M**
Total metal oxide	10.10	10.99
Total metal hydroxide	4.40	3.96

*The atomic amount has been naturalized*.

### Effect of Ethanol on Pitting Behavior in Immersion Tests

**Figure 2:**
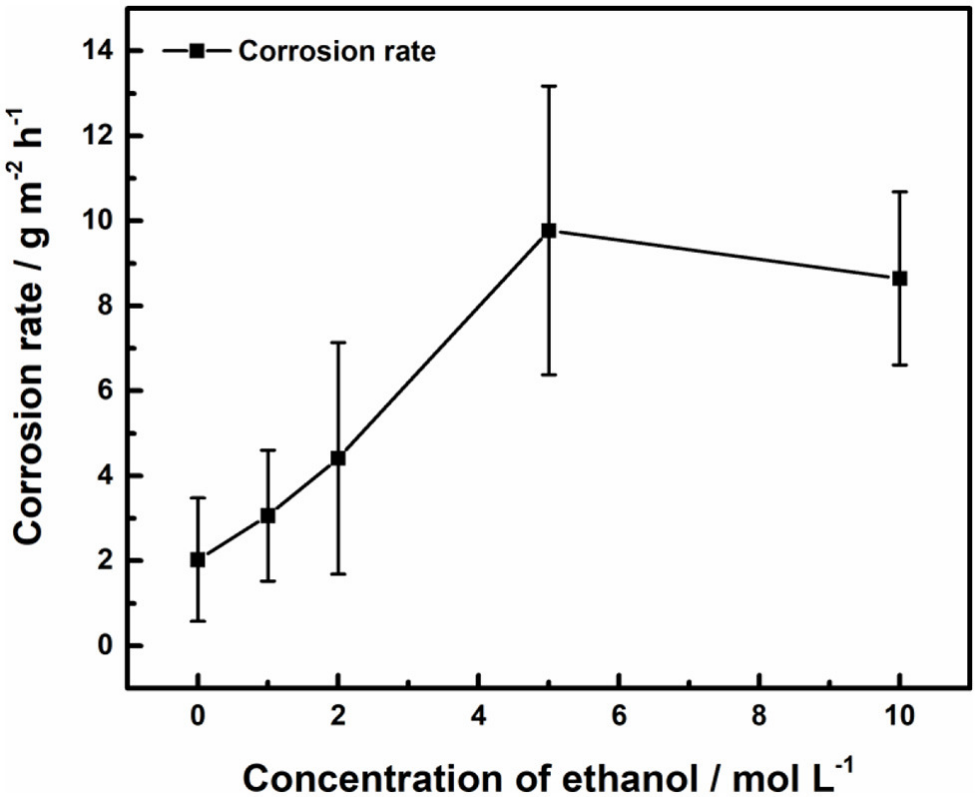
Effect of ethanol on corrosion rate of 316L samples.

The corrosion rate of 316L samples in acid ferric chloride solution with different ethanol concentrations is shown as Figure 2. With the increase of ethanol concentration, the corrosion rate of 316L samples first increased then decreased, indicating that ethanol should have both promoting and inhibiting effects on pitting. Corrosion rate reached 9.77 g m^−2^ h in 5 M ethanol, which was the highest case. While weight loss mitigated in 10 M ethanol, corrosion rate was still much higher than in lower ethanol concentration solutions.

**Figure 3:**
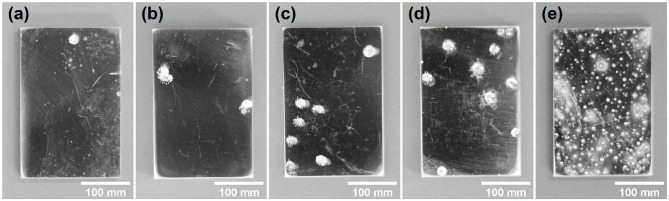
Pits pattern of the 316L samples after 24-h immersion tests in 0.3 M FeCl_3_ + 0.4 M HCl solutions with **(a)** 0 M, **(b)** 1 M, **(c)** 2 M, **(d)** 5 M, **(e)** 10 M ethanol.

**Figure 4:**
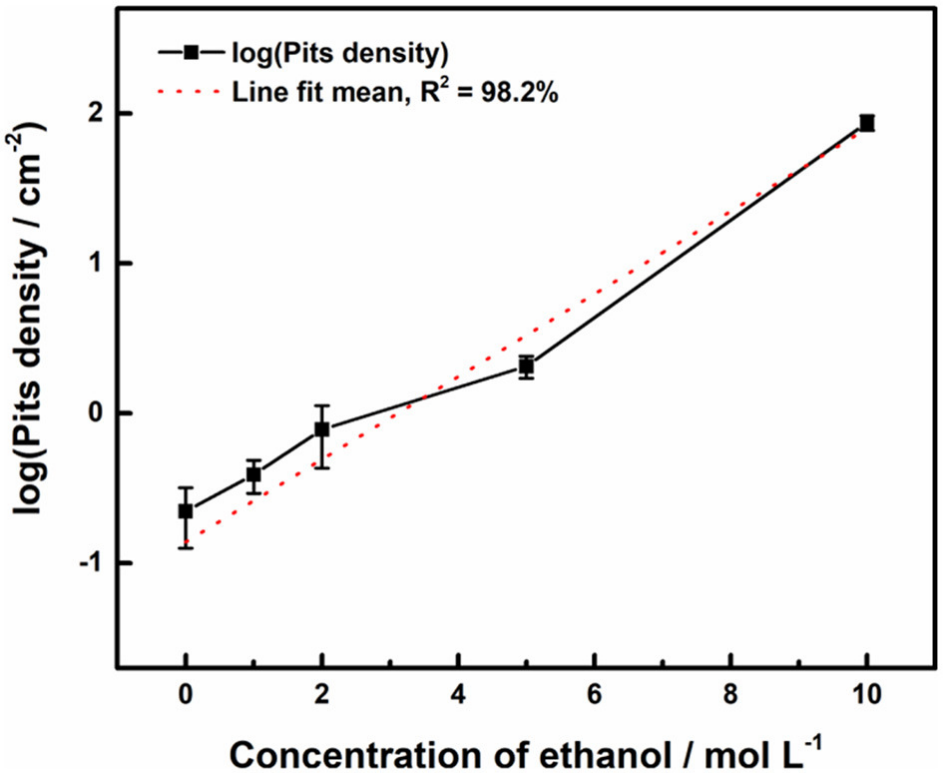
Effect of ethanol on number of pits initiated on the surface of 316L samples after immersion tests in 0.3 M FeCl_3_ + 0.4 M HCl solutions.

Figure 3 shows the surface profiles of samples immersed in acid ferric chloride solution with series of ethanol contents for 24 h. With the increase of ethanol concentrations from 0 to 5 M, the quantities of pits increased, and the surface area of the single pit changed little. For samples in 10 M ethanol, the surface of samples was densely covered with pits, and the areas of pits were much smaller, which was quite different from the pits pattern under 0 M ethanol to 5 M ethanol. As shown in Figure 4, the logarithm of the number of pits on the sample surface has a linear relationship with the concentration of ethanol. Such phenomenon can also be observed in methanolic solutions. In methanolic solutions, the change in pit density could be attributed to the oxidation from methanol to formic acid (Szklarska-Smialowska and Mankowski, 1982). However, similar mechanism cannot hold in ethanolic solution, since the oxidation from ethanol to acetic acid in acid medium takes place at 1.2 V (Tremiliosi-Filho et al., 1998), which is greater than the electrode potential of iron (III) toward iron (II) (771 mV vs. RHE). The mechanism of the effect of ethanol on the pits density will be discussed in section Discussion.

### Effect of Ethanol on Pitting Behavior of 316L Samples in Electrochemical Tests

**Figure 5:**
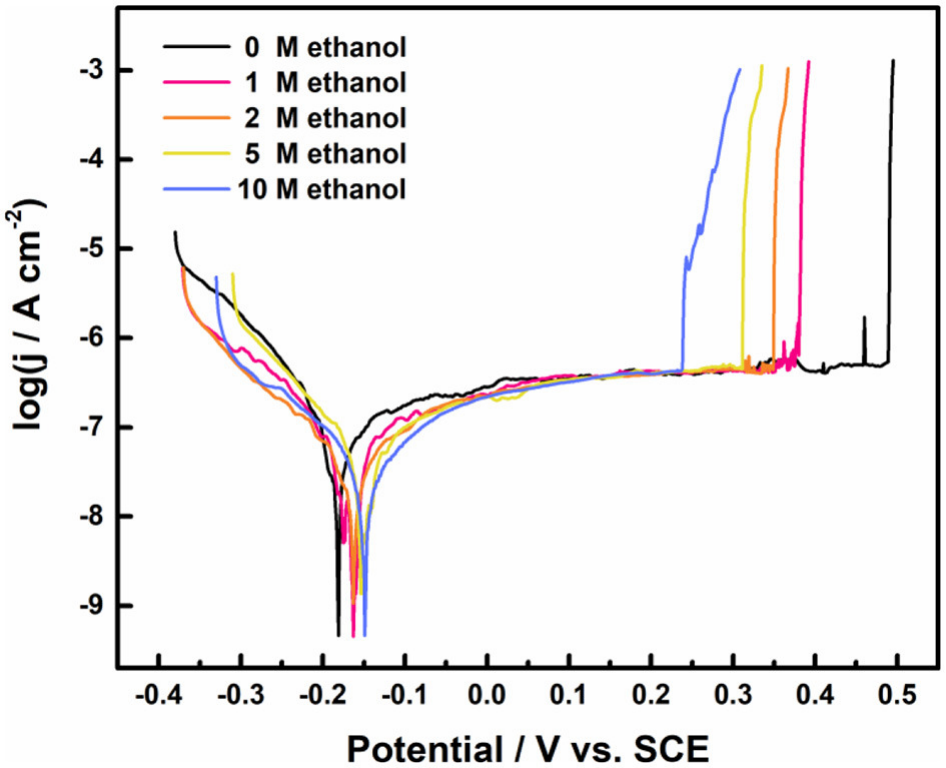
The potentiodynamic polarization curves of 316L stainless steel samples in 1 M NaCl solution with different ethanol concentration, terminated at 1 mA cm^−2^.

Figure 5 shows the polarization curves of 316L samples in 1 M NaCl solution with different ethanol contents at 30°C. The breaking potential gradually decreased from 489 to 239 mV with the addition of ethanol. In methanolic solutions, the dissolution of metal matrix in pits slows down due to the lower diffusion coefficient of metal cations (Ramgopal and Amancherla, 2012). Similar phenomenon occurred in the ethanolic solutions. At the rear stage after pits break down, the climb of current density got stuck in the ethanolic solutions. In 10 M ethanol, the current density fell obviously during climbing, which shared the same climb behavior in multiple tests.

The derived corrosion potential (*E*_*corr*_), breakdown potential (*E*_*b*_) and the average passive current density (*i*_*pass*_) values are listed in Table 3. *E*_*b*_ decreased significantly while *E*_*corr*_ and *i*_*pass*_ changed slightly as the concentration of ethanol increased. It proved that the pitting resistance of 316L did deteriorate with no obvious change in passivation film. In addition, the dispersion of *E*_*b*_ decreased with the increased ethanol content.

**Table 3:** Corrosion potential (*E*_*corr*_), breakdown potential (*E*_*b*_) and average passive current density (*i*_*pass*_) of 316L stainless steel samples in solutions with different ethanol content.

**C_**EtOH**_ (mol L^**−1**^)**	***E*_*corr*_ (mV)**	***E*_*b*_ (mV)**	***i*_*pass*_ (μA cm^**−2**^)**
0	−180 ± 22	492 ± 42	0.45 ± 0.02
1	−163 ± 19	369 ± 36	0.42 ± 0.01
2	−162 ± 20	350 ± 34	0.43 ± 0.01
5	−153 ± 15	321 ± 27	0.42 ± 0.01
10	−149 ± 19	240 ± 20	0.42 ± 0.01

**Figure 6:**
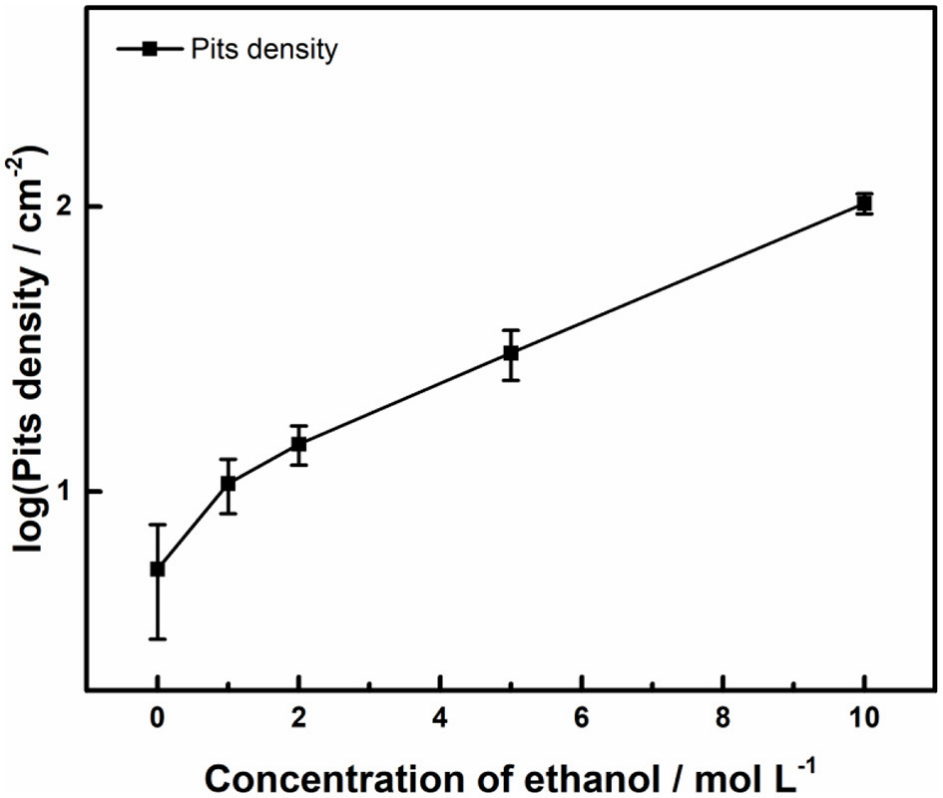
Effect of ethanol on number of pits initiated on the surface of 316L after polarization tests in 1 M NaCl solution with different ethanol concentrations at 30°C, terminated at 1 mA cm^−2^.

The pits pattern on the surface of 316L samples after potentiodynamic polarization tests was similar to the pattern in immersion tests. As shown in Figure 6, pits density increased as concentration of ethanol increased, but no exponential growth as in immersion tests occurred. Since the number of precursor sites on the surface of stainless steel is finite (Chen et al., 2020) and stable pits require enough time to incubate at lower overvoltage (Frankel et al., 1987), the reason for different trend from immersion experiments could be inadequate development time for activation of possible pits and deprivation of precursor sites.

**Figure 7:**
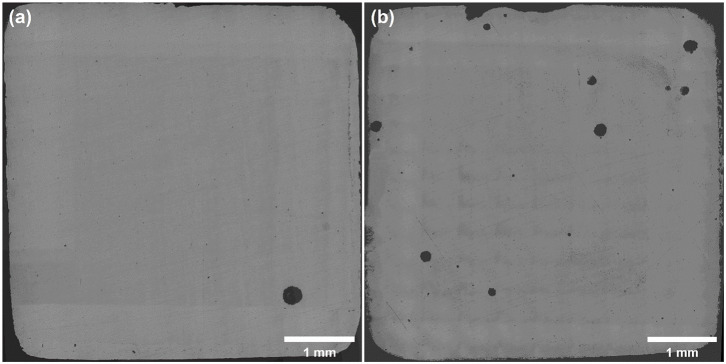
Panoramic optical micrograph of sample surfaces after polarization tests in 1M NaCl solution with **(a)** 0 M, **(b)** 10 M ethanol at 30°C, terminated at 1 mA cm^−2^. No ultrasound treatment was conducted.

As shown in Figure 7, panoramic optical micrographs of samples were taken and stitched after polarization tests. Many pits initiated on the surface of 316L samples after the polarization test in 1 M NaCl solution with 0 and 10 M ethanol terminated at 1 mA cm^−2^. Considering Figure 5, the dissolution charge after broken in 0 M ethanol was contributed by the single pit on the surface, which developed rapidly and generated higher current density. In 10 M ethanol case, the dissolution charge was formed by the joint contribution of several pits. While most of the pits failed to grow up to the size of the main pits, several pits developed to scale, which suggested that single pit failed to generate sufficient current density, and instead, more pits were initiated at a higher overvoltage to climb to the specified current density. It could be assumed that first single stable pit was generated at **E**_**b**_, and multiple pits were nucleated before the current density reached 1 mA cm^−2^.

**Figure 8:**
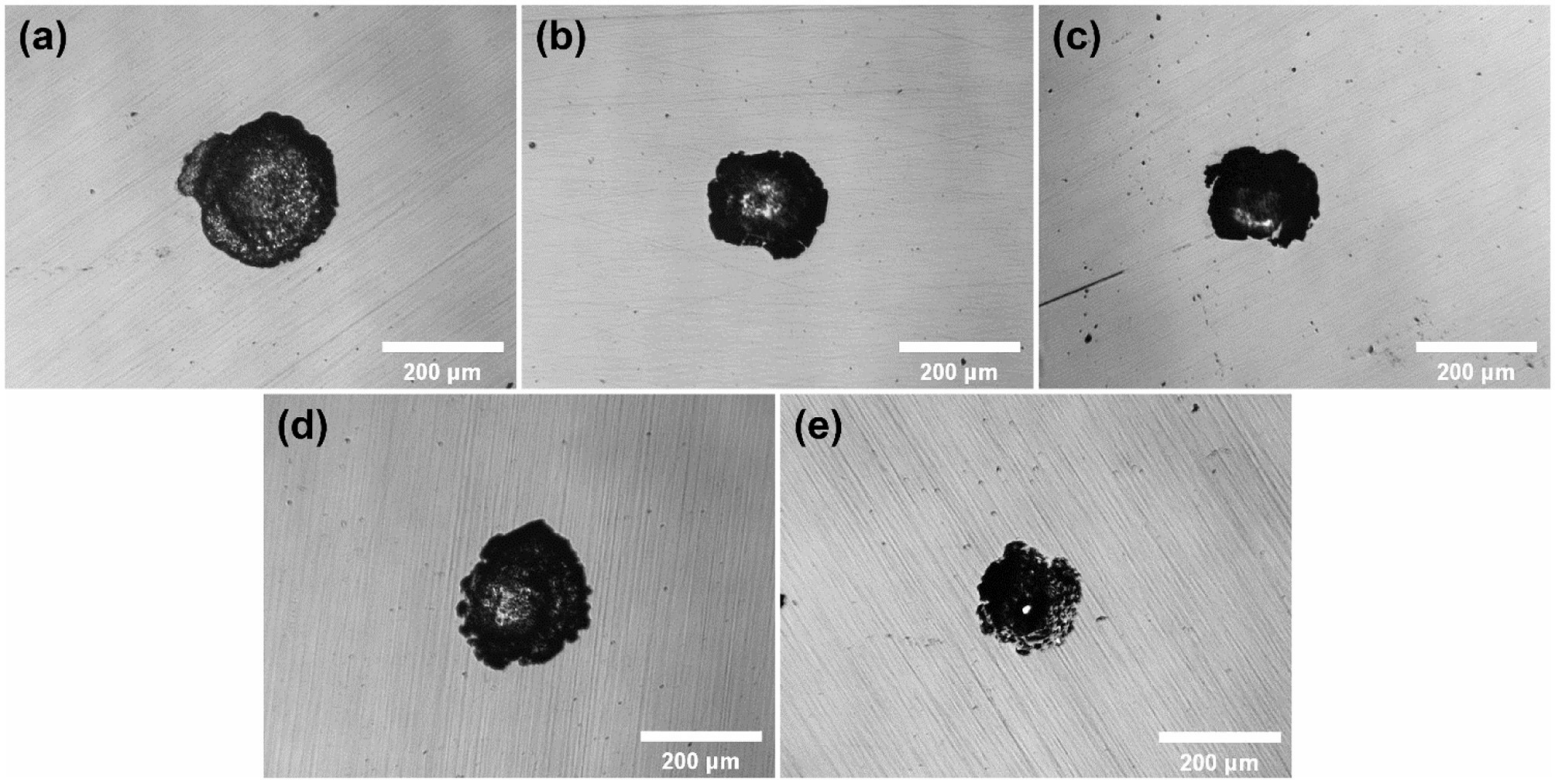
The optical micrographs of single pits on the surface of 316L samples after polarization tests terminated at 100 μA cm^−2^ in 1 M NaCl solutions with **(a)** 0 M, **(b)** 1 M, **(c)** 2 M, **(d)** 5 M, **(e)** 10 M ethanol.

Since mass pits and the different dissolution charge developed hindered the observation of the developing behavior of single pits under the same conditions, 100 μA cm^−2^ as a lower termination current density was applied to obtain the morphology of a single pitting on the sample surface. As shown in Figure 8, pits on the surface of 316L samples were limited to single. This confirms the previous assumption for first single stable pit at **E**_**b**_. It is noteworthy that the lacy cover occurred in 10 M ethanol. Lacy cover is generally considered to be affected by the aggressiveness of the solution in the pits. Distribution of metal cations in hemispherical pits controlled the dissolution of matrix near the surface of samples in aqueous environment (Ernst et al., 1997). In environment less aggressive, the inner surface of pits near matrix surface tends to oscillate between diffusion-control process and reaction-control process, and forms lacy cover.

**Figure 9:**
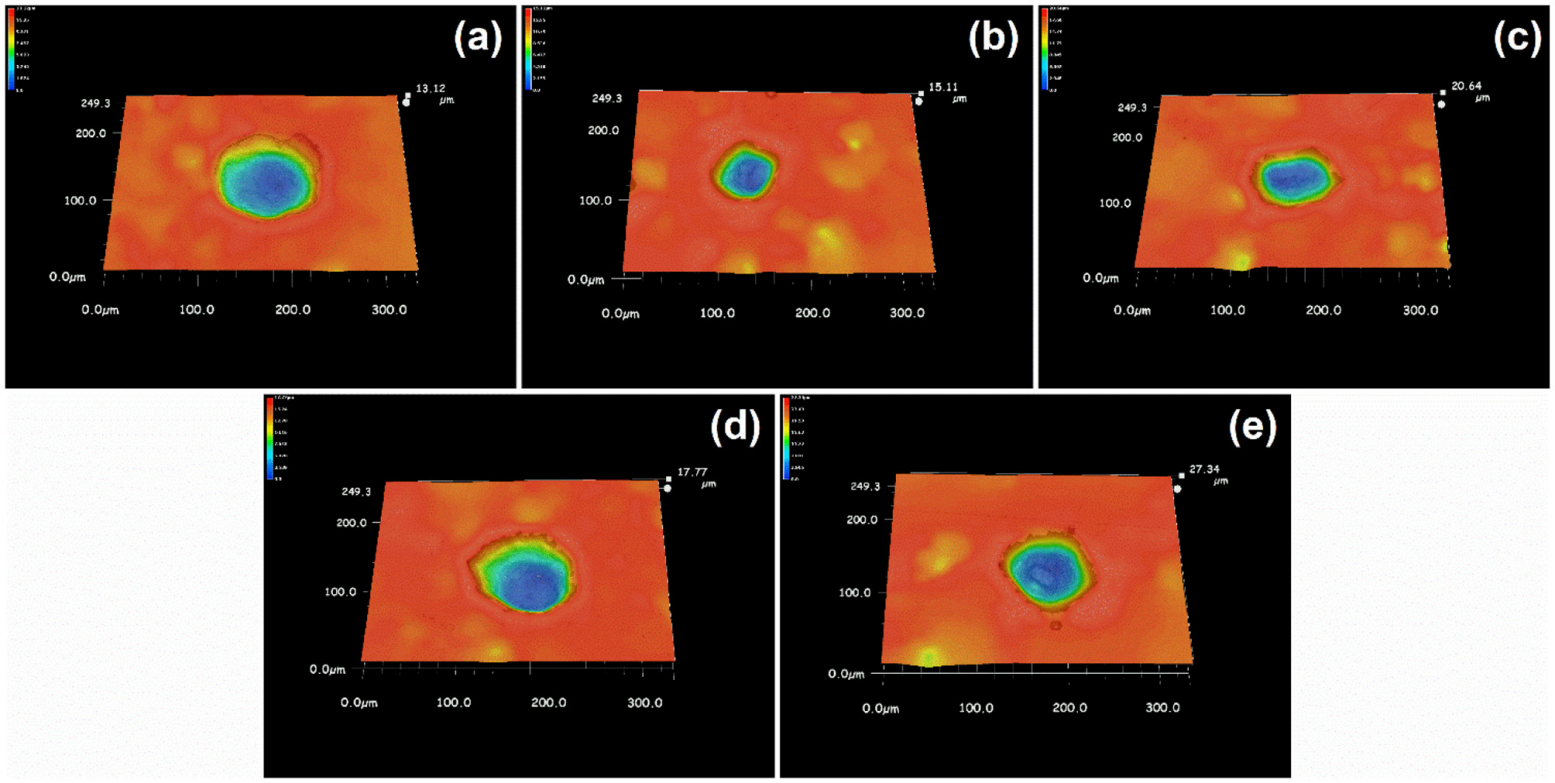
The 3D profile of single pits after ultrasound treatment after polarization tests terminated at 100 μA cm^−2^ in 1 M NaCl solutions with **(a)** 0 M, **(b)** 1 M, **(c)** 2 M, **(d)** 5 M, **(e)** 10 M ethanol.

**Figure 10:**
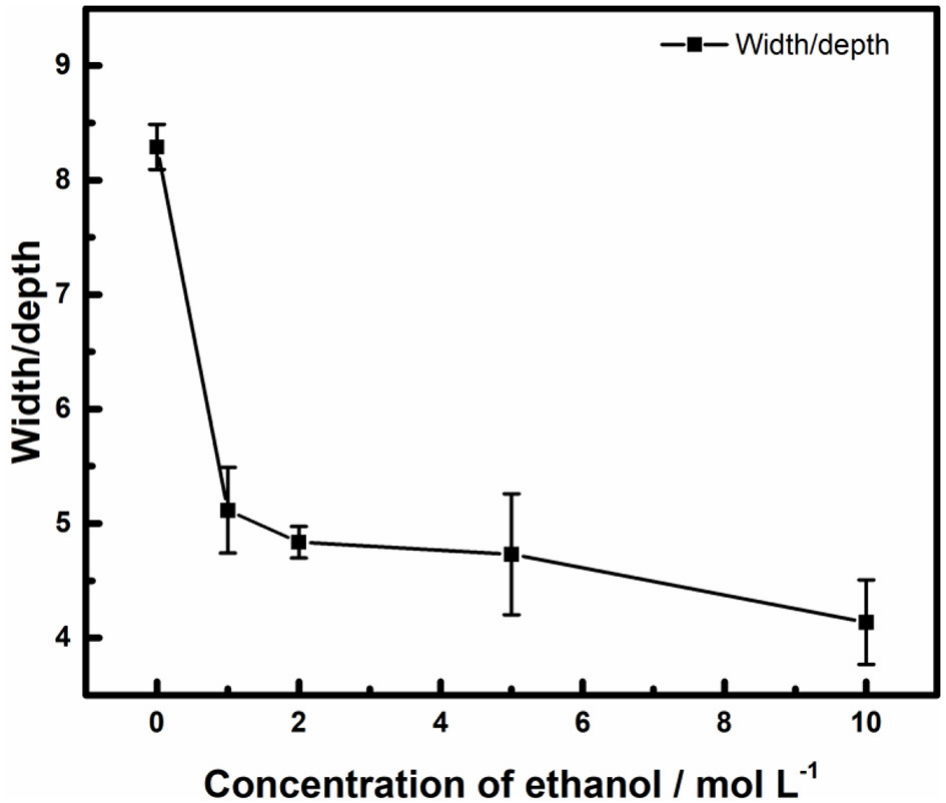
Effect of ethanol on the ratio of width and depth of pits on the surface of 316L samples after polarization tests terminated at 100 μA cm^−2^.

The 3D plots in Figure 9, shows that the edge of the pits on the surface of stainless steel owned a lower contrast in ethanolic solution where the dissolved surfaces were steeper. It reveals that the bottom of it. The ratio of width and depth of pits in different concentrations of ethanol was calculated and summarized in Figure 10. Once ethanol was present, the depth of the pits would be significantly deeper than in non-ethanolic environment.

## Discussion

Based on the experiment results above, the effect of ethanol on the pitting behavior of stainless steel was clear. In ethanolic solutions, the initiation of pitting was simplified, the development was suppressed, and the pits tended to be deeper. Since the development of stable pits is an electrochemical process controlled by metal cation diffusion (Frankel et al., 2017) and the higher viscosity of ethanolic solution (Khattab et al., 2012), the drop of ions diffusion rate was ascribed to the inhabitation of pitting development. However, the influence on pits initiation is not caused by the deterioration of the passivation film on the surface of 316L and the theory of alcohol electro-oxidation does not apply to ethanolic systems. Interpretations more suitable for this system need to be proposed.

**Figure 11:**
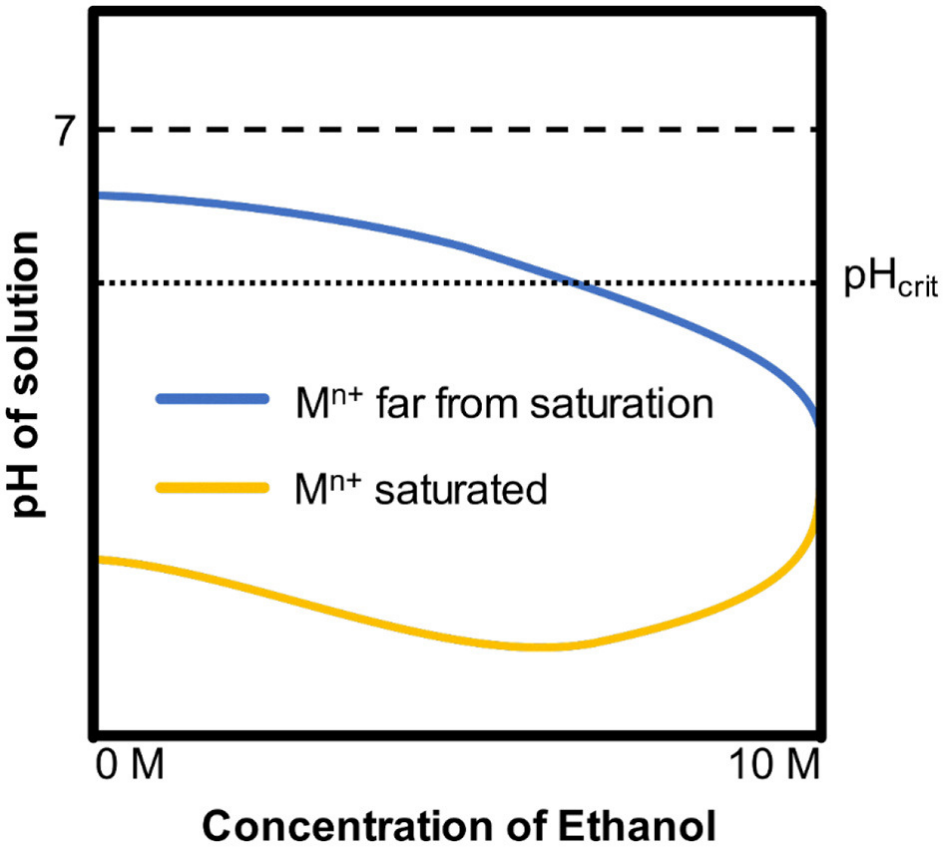
The schematic pH curves induced by hydrolysis of metal cations (Ni^2+^, Fe^2+^, Fe^3+^ etc.) in water with different ethanol contents.

Which has been neglected for a long time is that the introduction of ethanol results in the decrease of saturated metal cation concentration (M^n+^) at the bottom of pits. Ferrous ions are much less soluble in ethanol than in pure water (Pound, 1939). In addition, pH of solution has changed at the same metal cation concentration as the ethanol content increased. Ho et al. found that the decrease of the precipitation pH of the Ni(OH)_2_ as the ethanol content increased would enhance hydrolysis of metal cations, resulting in lower pH in ethanolic solutions (Ho and Van Zee, 2000). Based on those two works, schematic pH curves are illustrated as Figure 11, where pH_crit_ is the pH dissolves iron under a specified potential in the pourbaix diagrams for iron (Beverskog and Puigdomenech, 1996). When the concentration of M^n+^ is far from saturation, which is similar to the solution in pits during pitting initiation, the pH of solutions decreases as the ethanol content increases. When the M^n+^ is saturated, which is similar to the solution at the bottom of pits during pitting developments, as a result of the synergy effect of hydrolysis enhancement and solubility reduction, the pH of solutions first decreases then increases.

**Figure 12:**
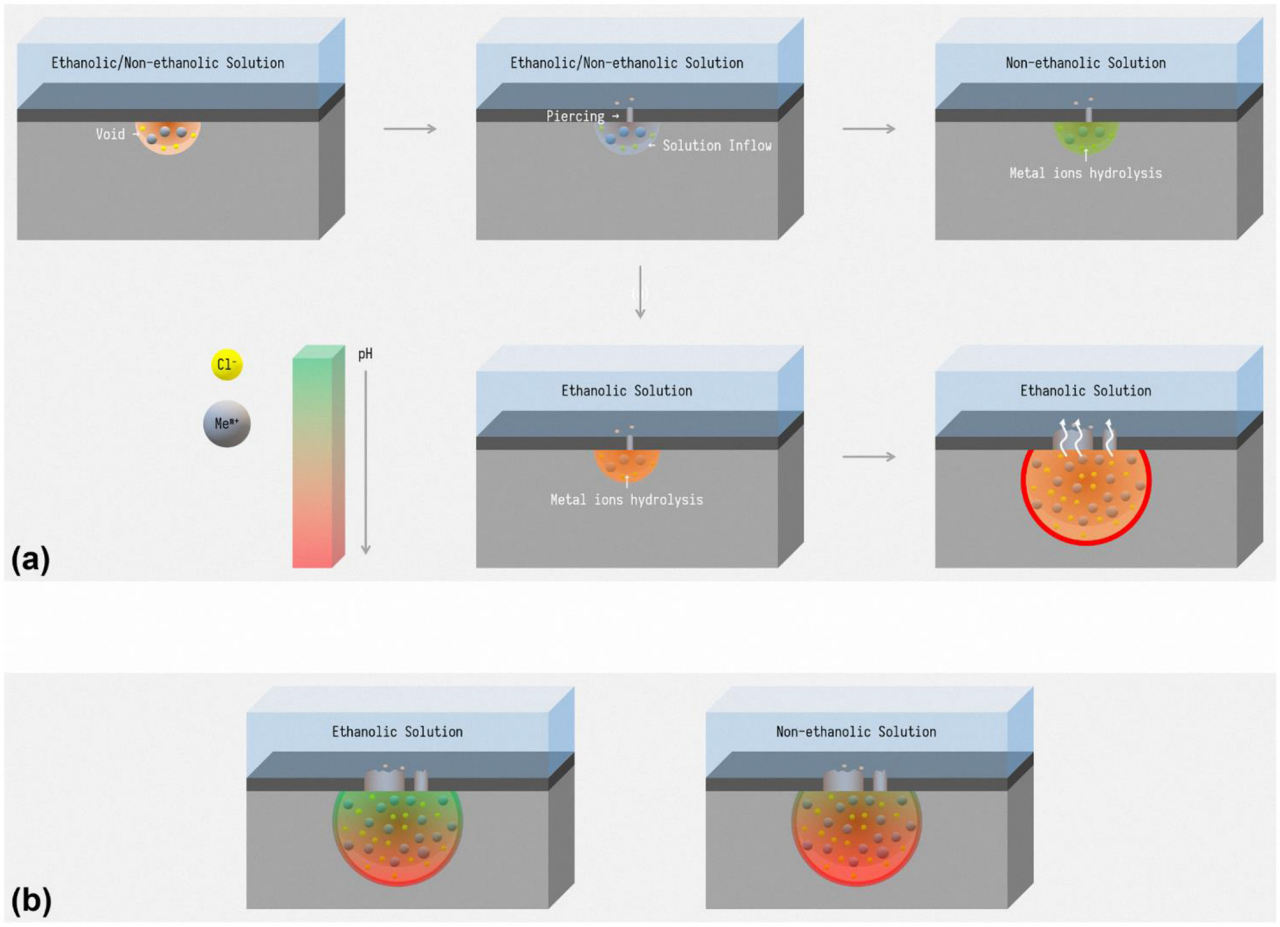
The schematic diagram of **(a)** pitting initialization, **(b)** pH distribution in pits, in ethanolic solutions and non-ethanolic solutions.

According to the schematic pH curves, schematic diagram of pitting initialization in ethanolic solutions and non-ethanolic solutions is described as Figure 12A. The metastable pits start with the injection of chloride ions and come into a void (Frankel et al., 1987). Since the performance of passive films changed slightly and ethanol does not affect the puncture behavior of chloride ions, the probabilities of this event are the same whether ethanol is introduced. Along with the expansion of the void, solutions inflow and contact the substrate and the difference of hydrolysis influences the result of pits initiation. The medium in ethanolic solutions have stronger acidity while pH of the medium in non-ethanolic solution failed to dissolve the matrix and the pits annihilated. As a result, pits are more easily activated in ethanolic solutions. Therefore, during the immersion tests, more pitting occurred on the surface of stainless steel in the ethanolic environment.

In addition, the synergy effect of metal cation solubility drop and hydrolysis enhancement is also ascribed to the morphology difference in ethanolic solutions. In stable pitting, a salt film forms on the inner surface of pits due to saturation of metal cations (Frankel, 1998). According to the yellow curve in Figure 11, the pH at the bottom of pits is higher in ethanolic solutions. As shown in Figure 12B, the concentration of metal cations at the bottom of pits is lower in high ethanol content than in non-ethanolic solution, resulting in dissatisfaction of pH to pH_crit_ near the surface. This leads to less dissolution at the top and more dissolution at the bottom of pitting in alcoholic solution, resulting in lacy cover and steeper pits. This feature also helped to understand the difference of pits pattern in 10 M ethanol immersion test. For sites in certain hemisphere pits, the current density of metal cations dissolution out of the pit could be approximated by Li et al. (2018).

(2) $$\begin{array}{r} i_{diff,site}=\frac{3nFDC_{site}}{2\pi r} \end{array}$$

where *n* is the average oxidation state of the metal cations, *F* is the Faraday constant, *D* is the effective diffusivity of the metal cations, *C*_*site*_ is the concentration of metal cations at the site, and *r* is the radius of the hemispherical pit. At the same time, the dissolution electric quantity of metal cations is given by

(3) $$\begin{array}{r} Q_{diff,site}=\int I_{diff,site}tdt=\int i_{diff,site}Stdt \end{array}$$

where *t* is the diffusion time range from pitting initiation to end of tests, and *S* is the effective area of pits, which considers the effect of lacy cover. Diffusivity of the metal cations decreases with the concentration of ethanol. The effect of lacy cover narrows the *S* value and slows down the rate of loss of ions from the pit. Under the suppress of diffusivity and *S* value, the size of pits in high concentration of ethanol are limited to a small scale.

## Conclusion

In this work, the effects of ethanol on the passive film, pitting behavior and pits morphology have been investigated with XPS analysis, immersion tests, potentiodynamic polarization tests and 3D microscope. The change in pitting behavior is due to hydrolysis enhancement and diffusion inhibition of metal cations in the ethanolic solutions. The effect of ethanol on the metal oxide content in the passive film on the surface of 316L stainless steel in borate buffer solution is slight. The corrosion rate of 316L stainless steel first increased then decreased while the initiation of pits is facilitated. The pitting potential of 316L stainless steel samples in 1 M NaCl solutions decreased from 489 to 239 mV with the gradual addition of ethanol. When the polarization curve was interrupted in the early stage of the pitting development to preserve single-pit pattern, the decrease in width-depth ratio of pits in ethanolic solutions could be observed.

## Data Availability Statement

The raw data supporting the conclusions of this article will be made available by the authors, without undue reservation.

## Author Contributions

YW drafted the manuscript. YS and LY reviewed and revised the manuscript. DC, ND, LL, YJ, and JL provided comments and suggestions. All authors contributed to the article and approved the submitted version.

## Conflict of Interest

The authors declare that the research was conducted in the absence of any commercial or financial relationships that could be construed as a potential conflict of interest.

## References

[B1] Abel J. Virtanen S. (2015). Corrosion of martensitic stainless steel in ethanol-containing gasoline: influence of contamination by chloride, H2O and acetic acid. Corros. Sci. 98, 318–326. 10.1016/j.corsci.2015.05.027

[B2] Beavers J. A. Gui F. Sridhar N. (2011). Effects of environmental and metallurgical factors on the stress corrosion cracking of carbon steel in fuel-grade ethanol. Corrosion 67, 025005–1–025005–15. 10.5006/1.3553341

[B3] Beverskog B. Puigdomenech I. (1996). Revised pourbaix diagrams for iron at 25–300°C. Corros. Sci. 38, 2121–2135. 10.1016/S0010-938X(96)00067-4

[B4] Bisht B. Bhandari H. Ruhi G. Gairola S. P. Dhawan S. K. (2017). Evaluation of an advanced self healing and highly durable corrosion protective epoxy coating modified with poly (Aniline-co-Pentafluoroaniline)/ZrO2 nanocomposite on mild steel. Curr. Smart Mater. 2, 130–145. 10.2174/2405465802666170707163408

[B5] Calabrese L. Bruzzaniti P. Proverbio E. (2018). Pitting corrosion of aluminum alloys in anhydrous ethanol. Mater. Corros. 69, 1815–1826. 10.1002/maco.201810125

[B6] Chen B. Sun Y. Cai D. Yao Q. Yin L. Wan Y. (2020). Use of the potentiostatic pulse technique to study and influence pitting behavior of 317L stainless steel. J. Electrochem. Soc. 167:041509 10.1149/1945-7111/ab7983

[B7] de Anna P. L. (1985). The effects of water and chloride ions on the electrochemical behaviour of iron and 304l stainless steel in alcohols. Corros. Sci. 25, 43–53. 10.1016/0010-938X(85)90087-3

[B8] Designation A. (2009). Standard Test Methods for Pitting and Crevice Corrosion Resistance of Stainless Steels and Related Alloys by Use of Ferric Chloride Solution. ASTM International.

[B9] Ekilik V. V. Grigor'ev V. P. (2002). Metal corrosion in organic and aqueous-organic media. Prot. Metals 38, 124–131. 10.1023/A:1014960915029

[B10] Ernst P. Laycock N. J. Moayed M. H. Newman R. C. (1997). The mechanism of lacy cover formation in pitting. Corros. Sci. 39, 1133–1136.

[B11] Ferreira E. A. Della Noce R. Fugivara C. S. Benedetti A. V. (2013). Influence of ethanol, acidity and chloride concentration on the corrosion resistance of AISI 316L stainless steel. J. Braz. Chem. Soc. 24, 397–405. 10.5935/0103-5053.20130052

[B12] Frankel G. S. (1998). Pitting corrosion of metals a review of the critical factors. J. Electrochem. Soc. 145, 2186–2198.

[B13] Frankel G. S. Li T. Scully J. R. (2017). Perspective—localized corrosion: passive film breakdown vs pit growth stability. J. Electrochem. Soc. 164:C180 10.1149/2.1381704jes

[B14] Frankel G. S. Stockert L. Hunkeler F. Boehni H. (1987). Metastable pitting of stainless steel. Corrosion 43, 429–436.

[B15] Gui F. Sridhar N. Beavers J. A. (2010). Localized corrosion of carbon steel and its implications on the mechanism and inhibition of stress corrosion cracking in fuel-grade ethanol. Corrosion 66, 125001-125001–12. 10.5006/1.3524831

[B16] Ho C.-H. Van Zee J. W. (2000). Effect of Ethanol and Temperature on the Hydrolysis of a Nickel(II) Ion in Ethanol–Water Solutions. Ind. Eng. Chem. Res. 39, 752–758. 10.1021/ie9702789

[B17] Hronsky P. (1981). Corrosion behavior of metallic materials in organic media containing hydrogen chloride. Corrosion 37, 161–170. 10.5006/1.3622160

[B18] Hronsky P. Duquette D. J. (1982). Pitting behavior of duplex 308L stainless steel in methanol/water/HCl solutions. Corrosion 38, 63–69. 10.5006/1.3577327

[B19] Hu E. Xu Y. Hu X. Pan L. Jiang S. (2012). Corrosion behaviors of metals in biodiesel from rapeseed oil and methanol. Renew. Energy 37, 371–378. 10.1016/j.renene.2011.07.010

[B20] Kahyarian A. Schumaker A. Brown B. Nesic S. (2017). Acidic corrosion of mild steel in the presence of acetic acid: mechanism and prediction. Electrochim. Acta 258, 639–652. 10.1016/j.electacta.2017.11.109

[B21] Kelly R. G. Moran P. J. (1990). The passivity of metals in organic solutions. Corros. Sci. 30, 495–509. 10.1016/0010-938X(90)90053-8

[B22] Khattab I. S. Bandarkar F. Fakhree M. A. A. Jouyban A. (2012). Density, viscosity, and surface tension of water+ethanol mixtures from 293 to 323K. Korean J. Chem. Eng. 29, 812–817. 10.1007/s11814-011-0239-6

[B23] Lee J. ye Li P. Lee J. Ryu H. J. Oh K. K. (2013). Ethanol production from *Saccharina japonica* using an optimized extremely low acid pretreatment followed by simultaneous saccharification and fermentation. Bioresour. Technol. 127, 119–125. 10.1016/j.biortech.2012.09.122 23131631

[B24] Lei L. Sun Y. Wang X. Jiang Y. Li J. (2020). Strategies to enhance corrosion resistance of Zn electrodes for next generation batteries. Front. Mater. 7:96 10.3389/fmats.2020.00096

[B25] Li T. Scully J. R. Frankel G. S. (2018). Localized corrosion: passive film breakdown vs pit growth stability: Part II. A model for critical pitting temperature. J. Electrochem. Soc. 165, C484–C491. 10.1149/2.0591809jes

[B26] Lou X. Singh P. M. (2010). Role of water, acetic acid and chloride on corrosion and pitting behaviour of carbon steel in fuel-grade ethanol. Corros. Sci. 52, 2303–2315. 10.1016/j.corsci.2010.03.034

[B27] Lou X. Yang D. Singh P. M. (2010). Film breakdown and anodic dissolution during stress corrosion cracking of carbon steel in bioethanol. J. Electrochem. Soc. 157, C86–C94. 10.1149/1.3269927

[B28] Newman R. C. (2008). Review and hypothesis for the stress corrosion mechanism of carbon steel in alcohols. Corrosion 64, 819–823. 10.5006/1.3279915

[B29] Okamoto G. Shibata T. (1970). Stability of passive stainless steel in relation to the potential of passivation treatment. Corros. Sci. 10, 371–378.

[B30] Pound J. R. (1939). The oxidation of solutions of ferrous chloride in alcohols. J. Phys. Chem. 43, 969–980. 10.1021/j150395a002

[B31] Radi P. A. Vieira A. Manfroi L. de Nass K. C. F. Ramos M. A. R. Leite P. (2019). Tribocorrosion and corrosion behavior of stainless steel coated with DLC films in ethanol with different concentrations of water. Ceram. Int. 45, 9686–9693. 10.1016/j.ceramint.2019.02.103

[B32] Ramgopal T. Amancherla S. (2012). Role of methanol on pitting of type 316 stainless steel. Corrosion 61, 1136–1144. 10.5006/1.3278150

[B33] Rocha G. J. M. Gonçalves A. R. Oliveira B. R. Olivares E. G. Rossell C. E. V. (2012). Steam explosion pretreatment reproduction and alkaline delignification reactions performed on a pilot scale with sugarcane bagasse for bioethanol production. Indus. Crops Prod. 35, 274–279. 10.1016/j.indcrop.2011.07.010

[B34] Saito H. Shibata T. Okamoto G. (1979). The inhibitive action of bound water in the passive film of stainless steel against chloride corrosion. Corros. Sci. 19, 693–708.

[B35] Samide A. Tutunaru B. Moantă A. Ionescu C. Tigae C. Vladu A.-C. (2015). A study of the surface protective layer formed on carbon steel in water-dioxane solution containing 0.15 M NaCl in presence of an azo dye with antimicrobial activity. Int. J. Electrochem. Sci. 10, 4637–4653. Available online at: https://apps.webofknowledge.com/full_record.do?product=UA&search_mode=GeneralSearch&qid=1&SID=6DDtpF7PvvEGB8narYU&page=1&doc=1

[B36] Samusawa I. Shiotani K. (2015). Influence and role of ethanol minor constituents of fuel grade ethanol on corrosion behavior of carbon steel. Corros. Sci. 90, 266–275. 10.1016/j.corsci.2014.10.020

[B37] Sarkar N. Ghosh S. K. Bannerjee S. Aikat K. (2012). Bioethanol production from agricultural wastes: an overview. Renew. Energy 37, 19–27. 10.1016/j.renene.2011.06.045

[B38] Shchukarev S. A. Tolmacheva T. A. (1968). Solubility of oxygen in ethanol—Water mixtures. J. Struct. Chem. 9, 16–21. 10.1007/BF00744018

[B39] Soriano C. Alfantazi A. (2016). Corrosion behavior of galvanized steel due to typical soil organics. Constr. Build. Mater. 102, 904–912. 10.1016/j.conbuildmat.2015.11.009

[B40] Sun Y. Liu X. Jiang Y. Li J. Ding J. Hu W. (2019). Recent advances and challenges in divalent and multivalent metal electrodes for metal–air batteries. J. Mater. Chem. A 7, 18183–18208. 10.1039/C9TA05094A

[B41] Szklarska-Smialowska Z. Mankowski J. (1982). The pitting of stainless steel in water-containing methanol. Corros. Sci. 22, 1105–1112.

[B42] Torkkeli J. Saukkonen T. Hänninen H. (2015). Effect of MnS inclusion dissolution on carbon steel stress corrosion cracking in fuel-grade ethanol. Corros. Sci. 96, 14–22. 10.1016/j.corsci.2015.03.002

[B43] Torsner E. (2010). Solving corrosion problems in biofuels industry. Energy Mater. 5, 42–48. 10.1179/147842209X12579401586726

[B44] Tremiliosi-Filho G. Gonzalez E. R. Motheo A. J. Belgsir E. M. Léger J.-M. Lamy C. (1998). Electro-oxidation of ethanol on gold: analysis of the reaction products and mechanism. J. Electroanal. Chem. 444, 31–39. 10.1016/S0022-0728(97)00536-6

[B45] Wang F. Chen G. Zhang N. Liu X. Ma R. (2019). Engineering of carbon and other protective coating layers for stabilizing silicon anode materials. Carbon Energy 1, 219–245. 10.1002/cey2.24

[B46] Wang J.-B. Wang J.-M. Shao H.-B. Zhang J.-Q. Cao C.-N. (2007). The corrosion and electrochemical behaviour of pure aluminium in alkaline methanol solutions. J. Appl. Electrochem. 37, 753–758. 10.1007/s10800-007-9310-8

[B47] Wang L. Gao H. Fang H. Wang S. Sun J. (2016). Effect of methanol on the electrochemical behaviour and surface conductivity of niobium carbide-modified stainless steel for DMFC bipolar plate. Int. J. Hydrogen Energy 41, 14864–14871. 10.1016/j.ijhydene.2016.07.037

